# Supplementary material for: Modeling glioblastoma heterogeneity as a dynamic network of cell states
Source: Mol Syst Biol. 2021 Sep 16;17(9):e10105. doi: 10.15252/msb.202010105 (PMC8444284; doi:10.15252/msb.202010105)
Supplement: Supplementary file 5 — Source Data for Figure 3 [file MSB-17-e10105-s001.zip › Figure3A_sourcedata/GSEA_3065/nosigdb_state2.GseaPreranked.1622718419215/RICHARDS_NEFTEL_CELL_2019_G1_S.html]

Details for gene set RICHARDS\_NEFTEL\_CELL\_2019\_G1\_S[GSEA]

|  || Dataset | state2 |
| Phenotype | NoPhenotypeAvailable |
| Upregulated in class | na\_pos |
| GeneSet | RICHARDS\_NEFTEL\_CELL\_2019\_G1\_S |
| Enrichment Score (ES) | 0.9594329 |
| Normalized Enrichment Score (NES) | 2.5721228 |
| Nominal p-value | 0.0 |
| FDR q-value | 0.0 |
| FWER p-Value | 0.0 |
Table: GSEA Results Summary

  

Fig 1: Enrichment plot: RICHARDS\_NEFTEL\_CELL\_2019\_G1\_S      
 Profile of the Running ES Score & Positions of GeneSet Members on the Rank Ordered List

  

| PROBE | GENE SYMBOL | GENE\_TITLE | RANK IN GENE LIST | RANK METRIC SCORE | RUNNING ES | CORE ENRICHMENT || 1 | HIST1H4C |  |  | 0 | 1.309 | 0.1095 | Yes |
| 2 | MCM7 |  |  | 13 | 0.626 | 0.1606 | Yes |
| 3 | PCNA |  |  | 16 | 0.595 | 0.2102 | Yes |
| 4 | RRM2 |  |  | 22 | 0.556 | 0.2562 | Yes |
| 5 | CKS1B |  |  | 23 | 0.554 | 0.3025 | Yes |
| 6 | DUT |  |  | 24 | 0.552 | 0.3487 | Yes |
| 7 | GINS2 |  |  | 37 | 0.506 | 0.3898 | Yes |
| 8 | CENPK |  |  | 44 | 0.488 | 0.4300 | Yes |
| 9 | ZWINT |  |  | 45 | 0.488 | 0.4708 | Yes |
| 10 | MCM3 |  |  | 51 | 0.464 | 0.5091 | Yes |
| 11 | CENPM |  |  | 54 | 0.460 | 0.5473 | Yes |
| 12 | TK1 |  |  | 56 | 0.456 | 0.5854 | Yes |
| 13 | GMNN |  |  | 64 | 0.435 | 0.6210 | Yes |
| 14 | RRM1 |  |  | 72 | 0.412 | 0.6547 | Yes |
| 15 | ASF1B |  |  | 76 | 0.407 | 0.6884 | Yes |
| 16 | MCM4 |  |  | 79 | 0.399 | 0.7216 | Yes |
| 17 | RAD51AP1 |  |  | 90 | 0.388 | 0.7529 | Yes |
| 18 | CDC45 |  |  | 95 | 0.377 | 0.7840 | Yes |
| 19 | CDCA5 |  |  | 101 | 0.373 | 0.8147 | Yes |
| 20 | FEN1 |  |  | 107 | 0.371 | 0.8452 | Yes |
| 21 | MELK |  |  | 133 | 0.332 | 0.8703 | Yes |
| 22 | TMEM106C |  |  | 162 | 0.303 | 0.8927 | Yes |
| 23 | AURKB |  |  | 163 | 0.303 | 0.9180 | Yes |
| 24 | MCM2 |  |  | 228 | 0.262 | 0.9332 | Yes |
| 25 | CCNE2 |  |  | 288 | 0.236 | 0.9467 | Yes |
| 26 | PKMYT1 |  |  | 342 | 0.220 | 0.9594 | Yes |
| 27 | RNASEH2A |  |  | 1579 | 0.082 | 0.8350 | No |
Table: GSEA details [plain text format]

  

Fig 2: RICHARDS\_NEFTEL\_CELL\_2019\_G1\_S: Random ES distribution      
 Gene set null distribution of ES for **RICHARDS\_NEFTEL\_CELL\_2019\_G1\_S**
